# Removal of Methylene Blue from Aqueous Solution by Mixture of Reused Silica Gel Desiccant and Natural Sand or Eggshell Waste

**DOI:** 10.3390/ma16041618

**Published:** 2023-02-15

**Authors:** Tatjana Juzsakova, Ali Dawood Salman, Thamer Adnan Abdullah, Rashed Taleb Rasheed, Balázs Zsirka, Rasha R. Al-Shaikhly, Brindusa Sluser, Igor Cretescu

**Affiliations:** 1 Sustainability Solutions Research Lab, Research Centre for Biochemical, Environmental and Chemical Engineering, Faculty of Engineering, University of Pannonia, P.O. Box 158, H-8201 Veszprem, Hungary; 2 Department of Chemical and Petroleum Refining Engineering, College of Oil and Gas Engineering, Basra University for Oil and Gas, Basra 61004, Iraq; 3 Chemistry Branch, Applied Sciences Department, University of Technology, Baghdad 10070, Iraq; 4 Research Group of Analytical Chemistry/Laboratory for Surfaces and Nanostructures, Center for Natural Sciences, University of Pannonia, P.O. Box 158, H-8201 Veszprem, Hungary; 5 Department of Prosthetic Dental Technology, Faculty of Health and Medical Technology, Al-Farahidi University, Al-Jadiriyah Bridge, Baghdad 10070, Iraq; 6 Faculty of Chemical Engineering and Environmental Protection, “Gheorghe Asachi” Technical University of Iasi, 73, Blvd. D. Mangeron, 700050 Iasi, Romania

**Keywords:** methylene blue removal, adsorption, low-cost adsorbent mixture, eggshell, sand, silica desiccant

## Abstract

The purpose of this work was to develop, characterize and test new low-cost materials suitable for removing methylene blue dye from water and wastewater by adsorption. The solid materials consisted of silica gel powder (SG), silica gel mixed with eggshell powder (SG-ES) and a mixture of silica gel with sand from the western Iraqi desert (SG-SI). The samples were milled by using an electrical mixer and a ball mill, followed by a drying step. In addition, desert sand was acid-treated in order to remove impurities. The structure and chemical composition of the samples were investigated by X-ray diffraction (XRD), a scanning electron microscopy technique equipped with an energy-dispersive X-ray spectrometer (SEM-EDX), a low-temperature nitrogen adsorption (BET) technique, thermo-analytical (TG/TGA) measurements and Fourier-transformed infrared spectroscopy (FTIR). The previously mentioned materials were tested to remove methylene blue from an aqueous solution. The adsorption experiments were monitored by ultraviolet–visible (UV-Vis) spectrophotometry and showed that SG and SG-ES gave promising results for the methylene blue removal from water. After 40 min of treatment of the aqueous solution containing 10 mg/L of MB at room temperature, the tested SG, SG-ES and SG-SI materials were found to have 86%, 80% and 57% dye adsorption efficiency, respectively. Taking into consideration not only the adsorption activity of the studied material but their availability, cost and concepts of cleaner production and waste minimization, the developed silica gel with eggshell can be considered as a good, cost-effective alternative to commercially available activated-carbon-based adsorbents. Different kinetic and isotherm models were fitted to the experimental results. A pseudo-second-kinetics-order model revealed high correlation fitting, while the Freundlich model was found to appropriately describe the adsorption isotherm. The thermal stability during the possible regeneration process of the SG-ES adsorbent mixture and its interaction mechanism with cationic dye was discussed.

## 1. Introduction

Water pollution caused by dyes has become a serious global issue in recent years, garnering widespread attention due to the production of over 100,000 different types of industrial organic dyes and pigments [1]. More than 10,000 dye compounds are being produced each year worldwide, resulting in about 7 × 10^8^ kg of dyestuffs and dye intermediates [2,3]. Furthermore, a lot of dyes are spilled into the natural environment during their manufacturing and practical use, causing a lot of problems for the environment [4]. Even at extremely low concentrations, the colour of dyes in water is easily observable [5]. Dyes can reduce the light permeability of water, thus leading to photosynthesis deterioration in water bodies [6,7]. Numerous industries, such as textile, plastic, rubber, paper, cosmetics, food and printing, release dye-containing effluents. These effluents are often carcinogenic or could have toxic impacts on the health of humans and other living organisms [8]. Some dyes have been reported to exhibit carcinogenic and mutagenic effects for organisms living in water [9]. Moreover, the global quality of water is affected [10], with wastewaters contaminated with dyes considered harmful to ecosystems, human health [11] and aquatic ecosystems [6,12]. Methylene blue (MB) is an organic cationic dye with an aromatic structure that is used in the textile industry for colouring silk and cotton [13,14]. It can permanently damage human and animal eyes by direct contact, as well as producing additional effects, such as nausea, vomiting, mental disorders, methemoglobinemia, allergic dermatitis, skin irritation and local burns [15,16].

Removing dyestuffs from water is critical, and a series of techniques have been developed in this respect, which are often classified as chemical, biological and physical procedures [12,17]. Examples of green procedures of these types include: adsorption, biodegradation, coagulation–flocculation [18], electrolysis [19,20] and photodecomposition [21,22]. However, the oxidation procedure is the most effective [23], allowing the complete decomposition of the pollutants. The biological processes degrade the organic molecules from wastewaters by using bioremediation systems, applying the microbial degradation or adsorption on biomass or fungal colonies. A drawback of the biological treatment is that it is a less effective and time-consuming process [24]. The chemical techniques, which are very efficient in treating polluted water, consist of the production of numerous by-products [24,25].

The adsorption techniques are also a widely applied strategy for an efficient water treatment approach [13,26]. They are recommended because of their low cost, facile operation and simple designs [15,26]. The key point of this method is the development of performant adsorbents. Adsorption can be either physisorption (characterized by weak intermolecular forces) or chemisorption (involving the formation of chemical bonding between the sorbate molecule and the sites on the surface) [27].

The literature indicates that dye pollutants can be successfully removed from an aqueous solution by adsorption on activated carbons derived from wastes [28]. Nanoparticles (particles smaller than 100 nm) have a high adsorption potential for removing dyes from water [29]. Finding new alternatives for manufacturing less expensive, economic and eco-friendly adsorbents for dyes removal [30,31] is always a challenge. Silica-rich materials contain numerous functional groups (-Si-OH), with a high capability for binding water contaminants through the formation of hydrogen bonds. An example is the use of diatomaceous earth (“bio-silica”) tested for the adsorption of organic dyes. This material is a siliceous biological sedimentary rock with a porous structure, consisting of 87–91% silicon dioxide, which is efficient in retaining organic dyes from aqueous media due to its large surface area and functional groups [32].

Several methods are cited for their adsorptive removal of methylene blue from water, including activated carbon [33], graphene oxide [34], green tea waste [35], etc. Many of these materials are sometimes expensive or difficult to acquire in convenient amounts. 

The use of cheap and available materials, such as silica gel [36], silica sand [37] and eggshells [38], has been studied alone in cationic dye removal but not in combination. 

The main purpose of this study was to investigate methylene blue water cleaning by adsorption using low-cost adsorbents containing two components, such as silica gel desiccant–natural silica sand (SG-IS) and silica gel desiccant–eggshell (SG-ES). The combination of individual IS and EG constituents may futher decrease the cost of materials while preserving silica gel adsorption property.

Silica gel is easily available from foods and nutrients containers and deciccant bags, while eggshell is a common kitchen waste based on calcite. Quartz is the main mineral of sand. Sand is a natural resource and can be found in huge amount in deserts and on beaches around the world. It is a low-cost and environmentally friendly non-toxic material. Due to these aspects, developing a practical setup for industrial wastewater cleaning with low cost in terms of initial investment, operation costs and efficiency is interesting from an economical point of view, especially in less-developed countries. 

The nature of the adsorbent material, and the dosage, contact time, initial dye concentration, temperature and pH of the solutions are the main parameters that affect adsorption performance. This research is mainly focused on development of new types of adsorbents and finding the optimum time for suitable pollutant removal efficiency.

The physico-chemical characterization of the individual constituents and their mixtures was performed by X-ray diffraction (XRD), scanning electron microscopy (SEM), energy-dispersed X-ray analysis (EDX), Fourier transform infrared spectroscopy (FT-IR), thermogravimetric analysis (TG/DTG and DTA) and low-temperature nitrogen adsorption (BET). Analyses regarding the compositon of the sample, effects of the initial dye concentration and contact time for the MB removal efficiency from aqueous solutions performed in this study were followed by kinetic isthoterm investigations and data fitting to various models. MB concentration in the solution was monitrored by ultraviolet–visible (UV-Vis) spectroscopic technique.

Based on the obtained charachterizations of samples and their adsorption behaviour, the MB adsorption mechanism and possible regeneration process for the silica gel–eggshell sample were disscussed.

## 2. Materials and Methods

### 2.1. Materials

Methylene blue (C_16_H_18_ClN_3_S) was provided by Scharlab S.L, Barcelona, Spain. Hydrochloric acid (HCl, ≥37%) and sodium hydroxide (NaOH, ≥96%) were purchased from VWR Chemicals BDH Co., Leuven, Belgium. Natural silica sand came from Iraq, used silica gel food bags and raw chicken eggshell were collected from a regular kitchen.

### 2.2. Preparation of the Adsorbents

#### 2.2.1. Silica Gel Powder (SG)

In this work, a desiccant-type material, silica gel, was studied, which is used to remove moisture from different food products during storage. Silica gel was drawn out from foods/nutrients lids and selectively collected and washed with distilled water three times to remove eventual impurities, before being activated by mixing for 5 h with HCl solution (3 mol/L) and finally rinsed with distilled water to neutralize the pH. The sample was dried at 100 °C for 4 h and ground for 15 min at 1000 rpm in an electrical grinder (Silver Crest-type device). Silica gel powder fraction sizes of <300 µm were used for study.

#### 2.2.2. Iraq Silica Sand Powder (SI)

Natural silica sand was obtained from the Ardma desert of western Iraq. The sand was first washed with sulfuric acid, then subjected to an acid leaching to lower the metal impurities, such as iron and aluminium oxides. The decrease in the aluminium content in sand resulted in an increase in its hydrophobicity. Subsequently, the product was washed with water, dried for 2 h at 120 °C and sieved in a shaking device to obtain a narrow fraction of average particle sizes of 300 µm. SI was next milled in a ball mill for 2 h at 250 rpm and sieved to separate the particles smaller than 300 µm. The powder product was dried again at a 120 °C for 2 h.

#### 2.2.3. Preparation of Eggshell Powder (ES)

Raw chicken eggshells obtained from a regular kitchen were quickly rinsed with distilled water to eliminate any egg residue from the shells, dried for 30 min at 100 °C to remove the remaining moisture, and milled in an electrical grinder to ensure a size reduction, before being introduced to the balls milling grinder for 5 h. The obtained powder was passed through a mesh of 300 µm to remove the larger clusters formed during the milling process. The last fraction was dried again in an oven at 100 °C for 30 min. Eggshell powder fraction size of <300 µm was used further.

#### 2.2.4. Preparation of Powder Mixtures

**Figure 1:**
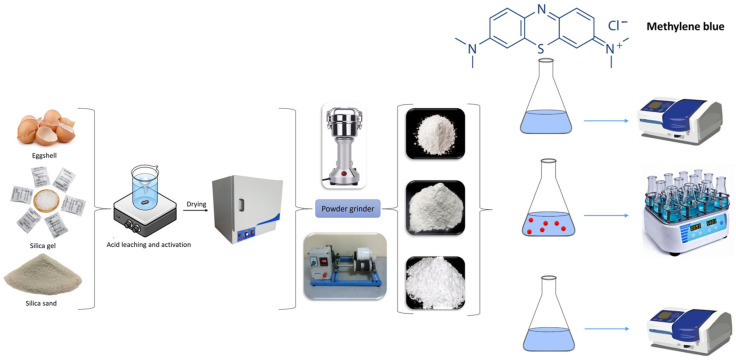
Adsorbents preparation and adsorption tests.

The preparation of silica gel and eggshell (SG-EG) and the silica gel and Iraq sand [39] (SG-SI) powder mixtures was performed by weighting equal amounts of the corresponding materials and homogenizing in the Silver Crest-type electrical grinder for 15 min. Afterwards, the powder mixtures were transferred to an oven for 1 h at 160 °C, ground in a ceramic mortar, and finally introduced to a muffle furnace at 200 °C. The preparation of adsorbents and the adsorption processes are shown in Figure 1.

### 2.3. Characterization

The identification of the solid crystalline phases from the samples was made by X-ray diffraction (XRD) on powder samples using a Philips PW3710 X-ray diffractometer (Almelo, The Netherlands) equipped with Cu-Kα radiation (λ = 0.1541 nm). The XRD patterns were recorded at room temperature over an angle range from 4 to 70° *2θ* at a scanning acquisition speed of 0.02°/s.

The morphology of the nanocomposites was studied by scanning electron microscopy (SEM) technique using a Thermo Fisher Scientific Apreo S LoVac SEM, (Brno, Czech Republic) operated at 2.0 kV for backscattered electron imaging, equipped with an energy-dispersive X-ray spectrometer (EDX), namely AMETEK’s Octane Elect Plus (Pleasanton, CA, USA).

The specific surface area, pore volume and pore size distribution in the 1.7–100 nm diameter ranges were determined from the N_2_ adsorption–desorption isotherms recorded at a low temperature of −196 °C using the Brunauer–Emmett–Teller (BET) [40] and Barret–Joyner–Halenda (BJH) models [41]. The samples (0.2–0.7 g) were first degassed under vacuum in a Micromeritics FlowPrep 060 facility at 160 °C for 2 h, then transferred to a Micromeritics 3 Flex 3500-type instrument (Norcross, GA, USA), before being placed under vacuum at 160 °C for further 4 h.

Infrared spectra were recorded using Bruker Vertex 70 equipment (Leipzig, Germany) with a Bruker Diamond ATR adapter by averaging 512 spectra at a resolution of 2 cm^−1^ using a room temperature DTGS detector. The powder samples were measured with further preparation by placing them onto a diamond ATR surface.

Thermo-analytical measurements were carried out using a Derivatograph PC (MOM, Budapest, Hungary). Powdered samples were loaded into open ceramic crucibles. TG, DTG and DTA curves were registered while heating the samples to 1000 °C (10 °C/min rate) in a static air atmosphere by using Al_2_O_3_ as DTA reference.

### 2.4. Adsorption Experiments

The methylene blue removal experiments from aqueous solutions were accomplished in batch mode at room temperature. The MB stock solutions of 20 mg/L were diluted with distilled water step by step to reach the proposed working concentrations of 10 mg/L. The pH of the solution was set to 7 with 0.1 N NaOH or 0.1 N HCl. In the experiment, 40 mL of MB solution was mixed with 10 mg of adsorbent. The MB removal efficiency was measured at established contact-time values, namely 5, 10, 20, 40 and 80 min. After each time interval, samples were taken from the organic dye concentration for analysis by UV-Vis Nanocolor spectrophotometer (Macherey-Nagel, Germany). The characteristic peak at 665 nm was used to determine the dye concentration value, as described in a previous work [29], on the basis of an initially traced linear Lambert–Beer calibration curve. 

The MB removal efficiency (RE) was calculated by dividing the eliminated dye concentration to the initial concentration value (Equation (1)) [29,42]:

(1) $$\mathrm{RE}\;\left(\%\right)=\left[\frac{C_{0}-C_{t}}{C_{0}}\right]*100$$

where:
RE—MB removal efficiency, %;
C_0_—initial MB concentration, mg/L;
C_t_—MB concentration at time t, mg/L.

The MB amount adsorbed at time t was calculated using (Equation (2)) [29,42].

(2) $$q_{t}=\frac{\left(C_{0}-C_{t}\right)}{m}*V$$

where:
V—solution volume, L;
m—adsorbent weight, g;
q_t_—MB adsorption capacity after time of contact of solution with the adsorbent, mg/g.

### 2.5. Kinetic Adsorption and Equilibrium Isotherms

The interactions between the sorbates and adsorbents are described by various mathematical models, such as equilibrium isotherms and adsorption kinetics. The adsorption equilibria explain the physicochemical processes involved in sorption and kinetic measures [43]. They also explain the transport mechanism of wastewater pollutants into the adsorbent, i.e., the mass transfer of the sorbate from the bulk solution to the surface of the sorbent, the internal diffusion of the sorbate to the adsorption site, and the overall adsorption process [44]. The kinetic models are relatively efficient when determining the rate at which the adsorbent efficiently removes the adsorbate, such as dyes. To ascertain the reproducible results, three different kinetic methods were applied to study the adsorption kinetics of pollutants on the sorbents, namely pseudo-first-order and pseudo-second-order reaction rates and intra-particle diffusion.

The pseudo-first-order reaction rate constant was calculated by Equation (3):

(3) $$\log\left(q_{e}-q_{t}\right)=\log q_{e}-k_{1}\frac{t}{2.303}$$

where q_e_ and q_t_ denote the amount of kerosene adsorbed (mg/g) at equilibrium and at time t (min), respectively, while k_1_ stands for the first-order rate constant (min^−1^).

A pseudo-second-order reaction based on the equilibrium capacity of adsorption is given by Equation (4):

(4) $$\frac{t}{q_{t}}=\frac{1}{k_{2}q_{e}^{2}}+\frac{t}{q_{e}}$$

where k_2_ denotes the equilibrium rate constant of pseudo-second-order adsorption (g/mg·min).

The third kinetic model, the intra-particle diffusion model based on the theory proposed by Weber and Morris, was used to identify the diffusion mechanism. The initial rate of intra-particle diffusion is expressed by Equation (5):

(5) $$q_{t}=K_{d}t^{1/2}+I$$

where K_d_ denotes the intra-particle diffusion rate constant (mg/g min^1/2^) and represents the intercept.

The Langmuir and Freundlich isotherm models were used to study the equilibrium isotherm, and the Langmuir model was also used to describe the monolayer’s homogeneous adsorption processes, showing that the adsorbent layer is stably located on the surface, with sites that are identical and no lateral interactions between the molecules. The Langmuir model is expressed by Equation (6):

(6) $$\frac{C_{e}}{q_{e}}=\frac{1}{q_{\max}}C_{e}+\frac{1}{q_{\max}b}$$

where C_e_ and q_e_ are the residual pollutant concentrations in solution (mg/L) and the amount of the pollutant adsorbed (mg/g) on the sorbent at equilibrium, respectively; q_max_ is the maximum amount of the pollutant per unit weight of sorbent (mg/g) and b is the Langmuir adsorption equilibrium constant (L/mg) related to the affinity between the sorbent and pollutant.

The Freundlich isotherm model is used to describe the non-ideal and reversible adsorption processes and is not limited to monolayer adsorption. Therefore, the Freundlich isotherm model is used to describe the multilayer adsorption, with a non-uniform distribution of the adsorption heat and sites of different affinities on heterogeneous adsorbent surfaces. The Freundlich isotherm model is expressed by Equation (7):

(7) $$\ln q_{e}=\ln K_{f}+\frac{1}{n}{\mathrm{lnC}}_{e}$$

where K_f_ and $\frac{1}{n}\;$ are the Freundlich adsorption constant (mg/g) (mg/L)^1/*n*^ and adsorption intensity, respectively.

## 3. Results and Discussion

### 3.1. X-ray Diffraction Results

**Figure 2:**
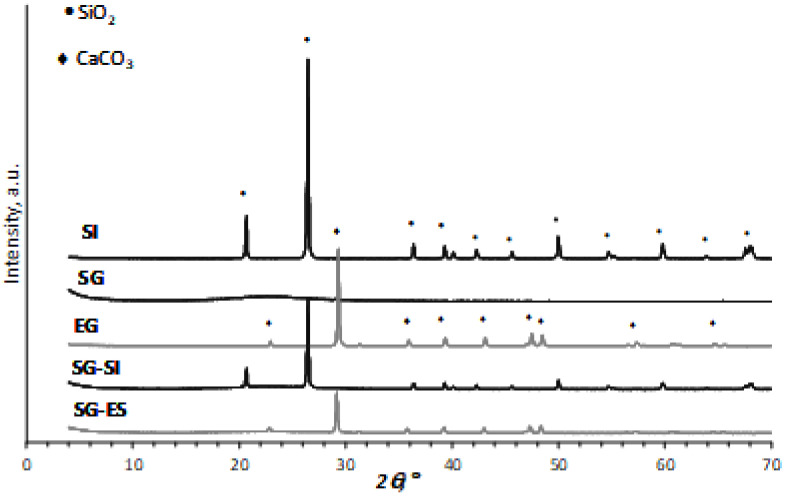
XRD patterns of the initial solids and the prepared adsorbents.

The XRD patterns for the SI, SG, ES, SG-ES and SG-SI are displayed in Figure 2. Mineral composition was identified using the following Powder Diffraction File cards: SiO_2_ (quartz: 00-046-1045), Al_2_O_3_ (corundum, 00-010-0173) and CaCO_3_ (calcite, 00-005-0586). The pattern of silica sand (SI) illustrates the existence of a high proportion of highly crystalline quartz, as shown by the (100) and (101) basal reflections corresponding to the maxima from 20.86° and 26.64° *2θ*, respectively. The minor amount of 2.45 wt% and 1.87 wt% Al_2_O_3_ was detected by EDX for the SI and SG-SI samples, respectively (see Table 1). However, no visible reflections assigned to corundum crystalline structure of alumina were detected, indicating its amorphous nature. 

Regarding the eggshell XRD pattern (EG), a significant amount of highly crystalline and almost pure calcite were identified on the basis of the reflections due to the (012) and (104) basal reflections appearing at 23.9 and 29.4° *2θ*, respectively. Silica gel (SG) is mainly amorphous, as seen from the extra-wide maximum ranging from 20 to 25° *2θ*.

The crystal nature of calcite and quartz remains unchanged after applying the intensive mechanical treatment. However, the mixing of SG and ES or SG and SI results in a decrease in the intensity of the reflections due to the decrease in the crystalline fractions of the individual constituents in a mixture with amorphous silica.

### 3.2. Scanning Electron Microscopic and Energy-Dispersive X-ray Analysis Results

**Figure 3:**
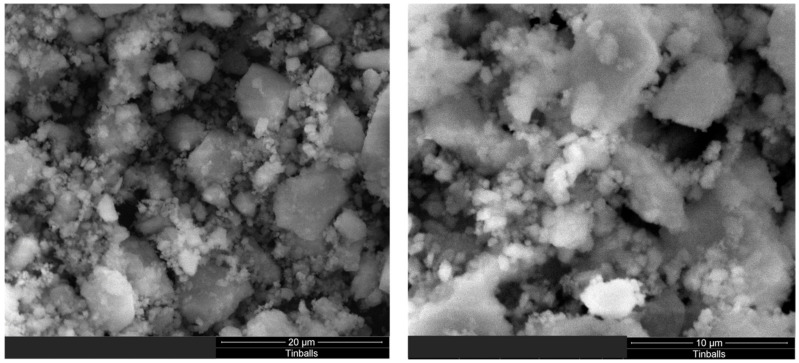
SEM images of the ES particles at scales of (**A**) 20 µm and (**B**) 10 µm.

**Figure 4:**
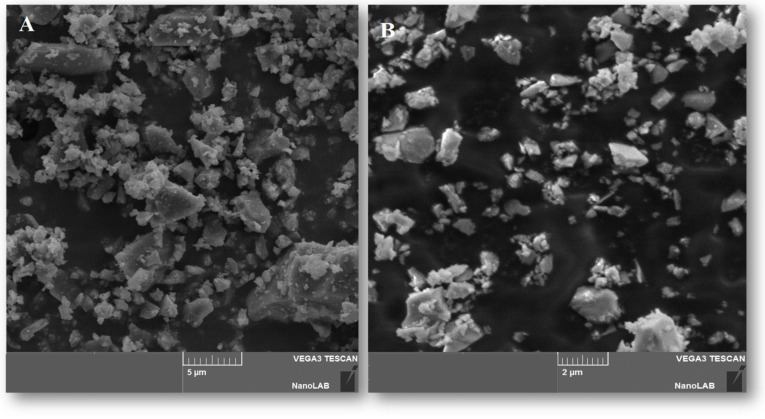
SEM images of the SI particles at scales of (**A**) 5 µm and (**B**) 2 µm.

**Figure 5:**
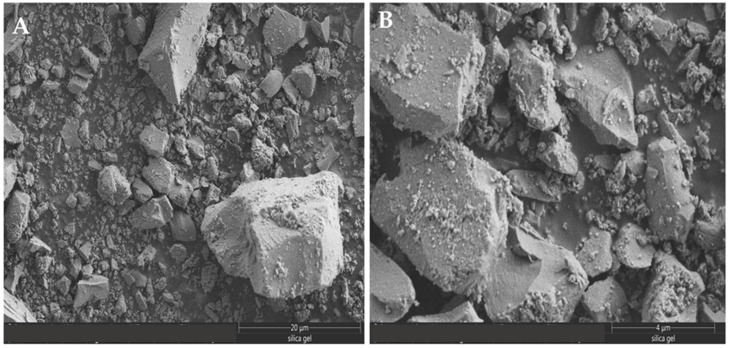
SEM images of milled SG particles at scales of (**A**) 20 µm and (**B**) 4 µm.

The SEM images of milled ES, SI and SG particles are presented in Figure 3, Figure 4 and Figure 5. The SEM images shown in Figure 3 and Figure 4 illustrate the morphology, texture, size and distribution of the ball-milled SI and EG particles, while Figure 5 shows the SEM images of the SG milled in an electrical grinder.

The ES particles have irregular shapes with rounded contours and different particle sizes (1–5 µm) due to the ball mill effect (Figure 3). On the contrary, the sand particles display a rugged shape and have more uniform sizes (0.5–2 µm) (Figure 4). The vigorous milling produces an important fraction of heterogeneous-sized particles of 4–20 µm, as well as irregular, rugged shapes (Figure 5).

**Figure 6:**
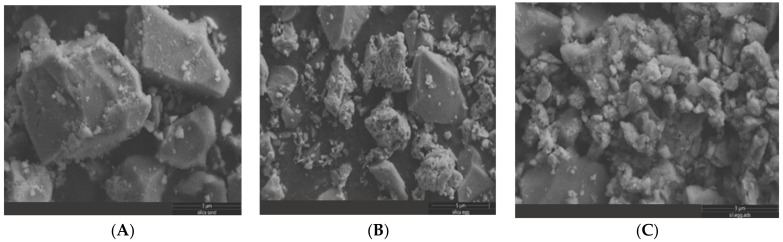
SEM images of mixtures of: (**A**) fresh SG-SI, (**B**) fresh SG-ES and (**C**) SG-ES after MB adsorption.

Figure 6 shows the SEM images of the mixed samples of fresh SG-SI and fresh SG-EG before and after MB adsorption. According to our records, the large-sized particles shown in Figure 6A,B belong to silica gel, while the small-sized particles belong to the silica sand and eggshell. 

After the grinding/milling processes, an aggregation of fine particles (<10 µm) can be observed, and the population of aggregated species was high for the EG and SI samples (Figure 3 and Figure 4) [45]. For solid, dried powders, the van der Waals weak attraction can lead to particle interaction and agglomeration [46]. After the MB adsorption on the SG-ES surface, the interaction between the mixed particles changed, and a higher degree of agglomeration was observed (Figure 6B,C). In MB aqueous solution, an electrostatic interaction might take place between the positively charged dye ions and negatively charged solid-surface species, causing the adsorbent particles to stick together. 

EDX results were recorded to determine the compositions of the studied samples (Figure S1 in the Supplementary Materials). The analysis was carried out on two areas of the specimens; the average measured compositions of the near-surface layer of the samples are also presented in Table 1. 

The main component for treated Iraq sand and silica gel is SiO_2_ (97–99%), and minor amounts of Al_2_O_3_ (0.75–2.5%) were detected. Possible other natural sand impurities, such as K_2_O, Fe_2_O_3_ and CaO were illuminated by acidic treatment and were not detected by EDX or XRD. The result is in agreement with an earlier reported study [39]. Both samples contain Na_2_O impurities in small amounts. The major oxides in the eggshell were CaCO_3_ (99%), accompanied by some SO_3_, MgO and SiO_2_ (amounts of less than 1 wt%). The major component of chicken eggshell is calcium in the form of calcite; however, minor inorganic elements, such as boron, chromium, copper, iron, iodine, manganese, sulphur, selenium, silicon, and zinc are expected to be present in the structure [47]. 

The composition of the mixed SG-EG sample estimated by EDX analysis was 1:1.6. The measured ratio was higher than the theoretical values used for preparation (1:1 mass ratio).

### 3.3. Fourier Transform Infrared (FTIR) Spectroscopy

**Figure 7:**
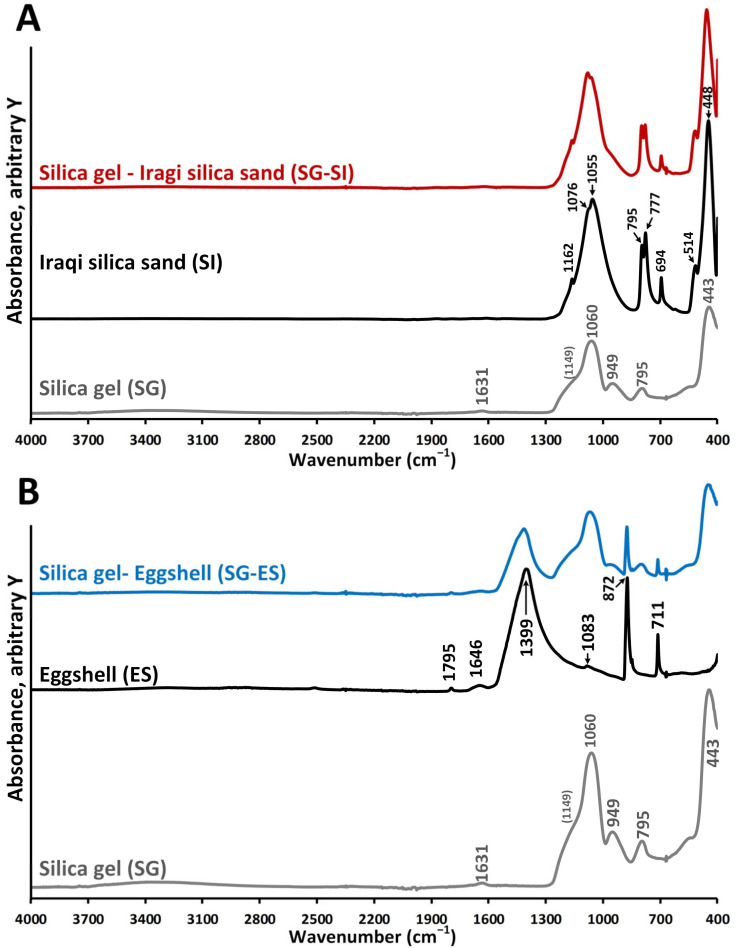
FTIR-ATR spectra of the two adsorbent mixtures and their constituents: (**A**) SG-SI, SI and SG (**B**) SG-ES, ES and SG samples.

The FTIR-ATR spectra of SG, ES and SI and the prepared adsorbents SG-ES and SG-SI are displayed in Figure 7A,B and Figure S2 in the Supplementary Materials. 

In harmony with the XRD results (Figure 2), the major constituent of SG is amorphous silica. The minor water content of the sample is indicated by the presence of a low intensity broad band between 3700 and 3000 cm^−1^, where the stretching bands of water (νHOH) are expected, as well as by the deformation band of the adsorbed water (δHOH) at 1631 cm^−1^. Although the stretching bands of hydroxylated silica (νSi-OH) groups were not observed [48,49], the band at 949 cm^−1^ could be assigned to the deformation of Si-OH hydrogen bonded with water molecules, as reported in [50]. The intense adsorption bands of the amorphous silica framework (Si-O-Si) were identified at 443 cm^−1^ (deformation), 795 cm^−1^ and 1060 cm^−1^ (stretching), with the latter having a shoulder around 1149 cm^−1^ [51].

The SI spectrum can be similarly interpreted as SG; however, the presence of ordered, crystalline quartz in the SI sample (Figure 2, XRD) results in the splitting of the Si-O-Si longitudinal/transverse-optical vibration modes. New bands were observed at 1162 cm^−1^, 1076 cm^−1^, 1055 cm^−1^, 777 cm^−1^ and 694 cm^−1^, originating from Si-O-Si stretching, while the bands at 694 cm^−1^ and 514 cm^−1^ were assigned to Si-O-Si deformations [49]. Overall, the IR spectra of SI confirm the presence of crystalline quartz [52,53].

In harmony with the XRD results (Figure 2), the eggshell sample predominantly consisted of calcite. Accordingly, the bands observed in the ES spectrum were associated with the vibrations of the carbonate anion (${\mathrm{CO}}_{3}^{2-}$): the band at 1399 cm^−1^ is due to the anti-symmetric stretching, while bending vibrations were observed at 872 cm^−1^ (out-of-plane) and 711 cm^−1^ (in-plane) [52,54]. This later band serves for the identification of calcite-CaCO_3_ [55], while the small intensity peak at 1083 cm^−1^ suggests the presence of a small amount of aragonite-CaCO_3_. The low intensity band at 1795 cm^−1^ could be assigned to a combination band of the carbonate ion [54].

The infrared spectra of the physically mixed adsorbents (SG-ES and SG-SI) can be regarded as the superimposition of the individual constituents (Figure S2 in Supplementary Materials) and are not discussed further.

### 3.4. Low-Temperature Nitrogen Adsorption

The detailed porous structure of an adsorbent is a key factor in both the adsorption’s overall performance and the diffusion role in the adsorption equilibrium settlement. The most convenient method for exploring the porous structure is the pure nitrogen adsorption performed at −196 °C, known also as BET adsorption. The adsorption data processed by applying BET an BHJ models can deliver various information, including total (S_BET_) and micropore (S_micro_) surface areas, total volume (V) for microspores of sizes between 1.7 and 100 nm and micropore volume (V_micro_) for pore sizes less than 2 nm. The pore size distribution and the average pore diameter (D_av_) could also be calculated. The information delivered by nitrogen adsorption analysis is displayed in Table 2. 

The surface area of the natural sand various from 1.8 to 15.0 m^2^/g depending on the place of origin [56,57]. The studied Iraqi sand contains a high amount of quartz, and its surface area (6.0 m^2^/g) was close to the value of 3.2 m^2^/g reported for quartz sand from Russia [58]. 

The SG desiccant sample has the largest surface area among the investigated samples (633 m^2^/g). Generally, commercially available silica gels used for drying and adsorbing moisture are porous materials with a large surface area of about 400–800 m^2^/g [59]. 

There are significant differences between the morphological characteristics of the initial materials, as well as between the prepared mixtures. The SG has a relatively high specific surface area (633 m^2^/g), while SI and ES have surfaces of two orders of magnitude lower (6 and 1 m^2^/g, respectively). 

After the addition of sand and eggshell to the silica gel, there were decreases in the surface area of 43% and 48% in comparison with the sample SG was observed for the SG-EG and SG-SI samples, respectively. This is an indication that both constituents are present in high amounts in the sample mixture. The decrease in the surface area in the case of mechanically mixed samples is associated with a decrease in the amount of porous silica gel by almost half by weight, as can be seen from Table 2.

The silica-gel-based mixtures still keep high specific surface values (330 and 360 m^2^/g, respectively, for SG-SI and SG-ES), recommending the use of the solids for the adsorption test. SG-ES has a higher surface area than the SG-SI mixture. This could be because the microporous structure is better preserved in the case of SG-SE, where the V_micro_ value for SG-ES is higher than for the SG-SI sample (Table 2).

The values of the total pore volumes vary in the same manner as the specific surface areas. The values of V of the mixed samples are also higher than the average calculated from the individual values of the components. The average pore size values for sand (28.1 nm) and eggshell (35.5 nm) are higher than for silica gel (2.3 nm) and the mixed samples (2.8–3.1 nm). The pores defined between the mixed particles are rather narrow, with a silica gel origin. The high values of the specific surfaces areas of the mixed samples and the narrow pores are a preamble for the good adsorption potential of the methylene blue molecule, which is well accommodated in the available pore space. According the literature data, the length of a methylene blue molecule is around 1.45 nm [60], and the width is approximately 0.95 nm [61].

### 3.5. Thermogravimetric Analysis Results

**Figure 8:**
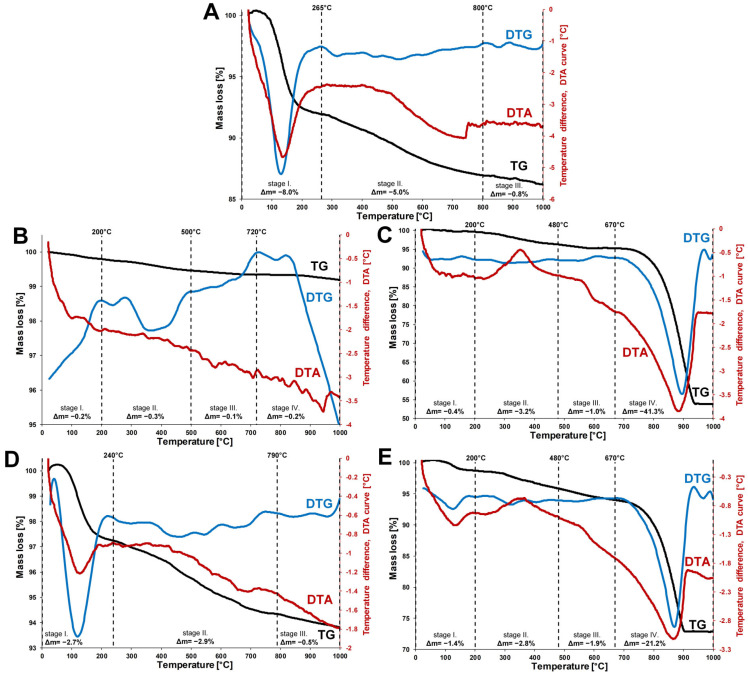
Thermo-analytical curves (TG/DTG and DTA) of the samples: (**A**) SG, (**B**) SI, (**C**) ES, (**D**) SG-SI, (**E**) SG-ES.

The thermo-analytical investigations results are presented in Figure 8 and Table S1 in the supporting information. The SG sample shows a first mass loss step up to 265 °C (Δm = −8%) (Figure 8A). This major endothermic mass loss is related to the dehydration of the hygroscopic SG, while the relatively high end-temperature (265 °C) is indicative of the presence of strongly bound adsorbed water. The mass loss in the second stage (265–800 °C, Δm = −5%) is mostly related to the elimination of chemisorbed water, with a possible contribution from the *dihydroxylation* of the silica surface [49,62]. The minor mass loss observed above 800 °C (Δm = −0.8%) can be assigned to the dihydroxylation of hydroxyl groups from the amorphous silica surface [63]. 

SI shows only minor mass loss upon heating to 1000 °C (∑Δm = −0.8%) (Figure 8B). The first mass loss stage indicates the negligible water content of the sample (20–200 °C, Δm = −0.2%). The residual organic carbon constituents from natural sand samples are expected to be lost in an exothermic reaction above 200 °C, as mentioned in [62]. For the SI sample, no exothermic effect could be identified above 200 °C, indicating that no residual organic matter was present in the sample due to the successful removal of organic impurities by sulfuric acid during sample preparation. The three minor mass loss steps from 200 °C up to 1000 °C can be related to the elimination of the surface hydroxyl groups from quartz [49,64,65], as well as the minor amounts of alumina pollutant/impurity [66,67] from the sample via high-temperature dehydration and dihydroxylation.

The ES sample shows a minor mass loss in the dehydration stage (Δm = −0.4%), indicating the low amount of surface adsorbed water (Figure 8C). Poultry eggshells can be regarded as bio-ceramics, and are generally constituted of 94–96% CaCO_3_ (calcite), with the rest being the organic matrix and various trace elements [68,69]. Apart from the removable shell membrane, the organic matrix is fused with parts of the inorganic shell [69] and is not expected to be removed via the pre-treatment of the sample. The oxidative removal of the organic constituents from the eggshell is observed as an exothermic mass loss during the second stage (200–480 °C, Δm = −3.2%). The thermal decomposition of calcite is expected above 600 °C [70,71], and hereby is observed from 670 °C to 1000 °C in a major mass loss step (Δm = −41.3%). The calcite content of the ES can be estimated from this thermal process ($CaCO_{3}\to CO_{2}+CaO$) utilizing the mass of the liberated CO_2_ (see SI-Y1 in the Supplementary Materials). The CaCO_3_ content of the dry sample is estimated to be 94.3%, which is similar to results reported in the literature [68,72].

Since the two adsorbent mixture (SG-SI and SG-ES) samples are physical mixtures, their thermograms can be regarded as an averaged superposition of the individual constituents (Figure 8D,E). In the case of SG-ES, the calcite decomposition step (670–1000 °C, Δm = −21.2%) can be used for the evaluation of the CaCO_3_ content. The carbonate content of the dry sample was estimated to be 48.90% (see SI-Y3 in Supplementary Materials). Considering the calcite content of the ES (94.3%), this would indicate that the dry SG-ES sample consists of 51.85% of the ES. (Table S1 in the Supplementary Materials). This value lies between 62% and 43%, which is determined by EDX (Table 1) analysis and supported by morphological measurements (Table 2).

### 3.6. Methylene Blue Adsorption from Water Solution

An MB adsorption study was carried out on SG and the SG-SI and SG-EG composites. The contact time between the solution and the sorbents varied between 0 and 80 min (sampling time values 5, 10, 20, 40 and 80 min). For the preliminary tests, the initial MB concentration was 10 mg/L, the solution volume was 40 mL and the adsorbent dose was 10 mg (corresponding to 0.25 g/L). The experiments were run at room temperature.

The adsorption of ionic dyes depends on the pH of the solution to be treated. The pH solution was set to 7 since at a pH close to the neutral region the optimum uptake of pollutant was expected [38]. In a more acid region, the cation dye competes with H^+^ ions on the solid surface of the adsorbent and, hence, the removal of MB is lower. It was observed that with increasing pH from 3.2 to 8 increases the negative charge density on the surface and results in an enhancement in the cationic MB [73]. 

**Figure 9:**
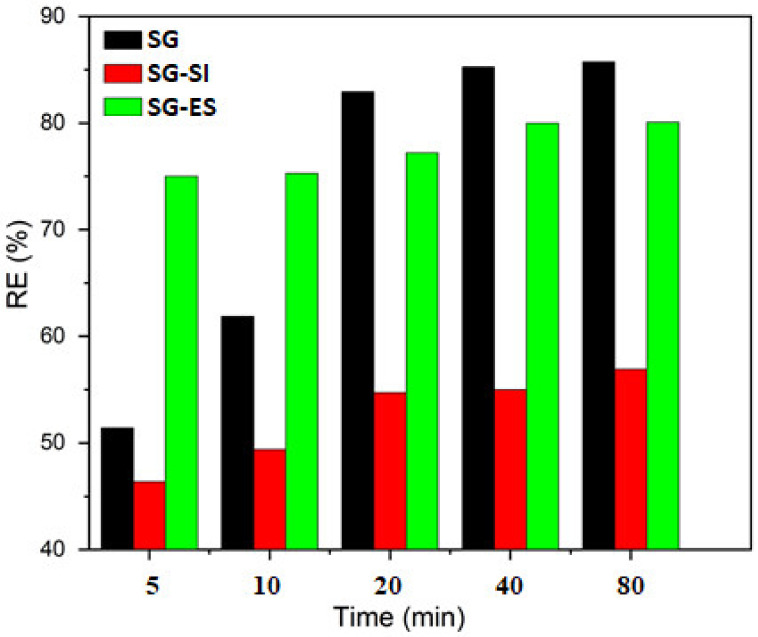
MB removal efficiency with time on SG, SG-SI and SG-EG samples (C_MB_ = 10 mg/L, V = 40 mL, m_ads_ = 10 mg).

The MB adsorption results are shown for silica gel and silica-gel-based samples. The results displayed in Figure 9 indicate the very different features of the adsorption on the previously mentioned materials. The SG and SG-EG composites were much more efficient than the SG-SI composite, both in terms of removal efficiency and rate of adsorption. The composite efficiency was even better than the pure SG, since the same adsorbent dose includes around one half SG and one half ES. The adsorption equilibrium seems to settle after approximately 40 min, with the removal efficiency reaching 86% on SG and 80% on the SG-ES mixture.

The adsorption capacities of the three samples were calculated in mg of dye per g adsorbent and followed the trend: SG (34.3 mg/g) > SG-SE (32.0 mg/g) > SG-SI (22.8 mg/g). 

The better adsorption capacity of the SG and SG-EG samples in comparison with SG-SI is probably related in part to the lower specific surface areas and free pore volumes, as well as to the lack of hydrophilic OH groups, as highlighted by the absence of the band from 3700 to 3000 cm^−1^ in the FT-IR spectrum (Table 2, Figure 7). Additionally, new adsorption species (discussed later in the text) are formed on the adsorption surface of SG-EG, such as HCO_3_^−^ and CO_3_^2−^ in the presence of eggshell material [38,74]. The formation of those active sites has a favourable effect on MB removal from the solution.

The MB adsorption capacity results achieved in this work show that the performance/adsorption activity of our composites is moderate in comparison with the literature data (Table 3). Table 3 shows that diverse solid waste and natural-material-based adsorbents displayed a broad range of dye adsorption capacity, from 2 to 100 mg/g [37,38,57,75,76,77]. These q_t_ values are lower in comparison with other commercial adsorbents, such as activated-carbon-based adsorbents (q_t_ ≈ 245 mg/g [78]), which are widely used in wastewater treatment. Furthermore, the cost of commercial adsorbent suitable for methylene blue removal, e.g., Norit A Supra Eur Usp (BET 1700 m^2^/g), is high, at about EUR 655 per 1 kg. The cost of SiO_2_ desiccant and sand (50–70 mesh) on the price list of the Sigma Aldrich chemical distribution company in Europe is about EUR 47 and EUR 65 per kg, respectively [79]. Therefore, the total price, including the cost of raw materials, chemicals and energy required for SG, SG-EG, and SG-IG preparation, would five to seven times lower. The cost of the proposed preparation samples could be further lowered if waste/natural materials are utilized: eggshell from kitchen waste, natural sand and silica desiccant bags.

It can be seen from the already studied systems listed in Table 3 that adding silica-based material to sand significantly improved its adsorption capacity from the 2.5–12 mg/g reported [37,57] to the 34 mg/g in this work. In our case, for a system containing eggshells, significantly lower adsorption properties were observed compared with the reported work [38]. The observed discrepancies in the results of the two groups may be related to the different experimental conditions used (adsorbent dose, MC concentration, treatment/adsorption time, laboratory equipment setup, etc.).

**Table 3:** Comparison between the removal efficiency and adsorption capacities for the MB dye removal of various systems, including this work.

Adsorbent Material	Initial Concentration, mg/L	RE, %	qt, mg/g	Ref.
diatomite	100.0	100.0	101.10	[75]
eggshell + membrane	1000.0	>95.0	94.90	[38]
brown peat	800.0	>93.0	24. 27	[76]
Sahara desert sand	13.1	90.0	11.98	[57]
sand	100.0–900.0	>99.0	2.50	[37]
coal fly ash (zeolite)	6.4	71.0	1.85	[77]
SiO_2_ desiccant (SG)	10.0	85.7	34.30	this work
SiO_2_ desiccant + eggshell (SG-EG)	10.0	80.1	32.00	this work
SiO_2_ desiccant + treated sand (SG-SI)	10.0	56.9	22.8	this work

### 3.7. Kinetics and Isotherm Models

**Figure 10:**
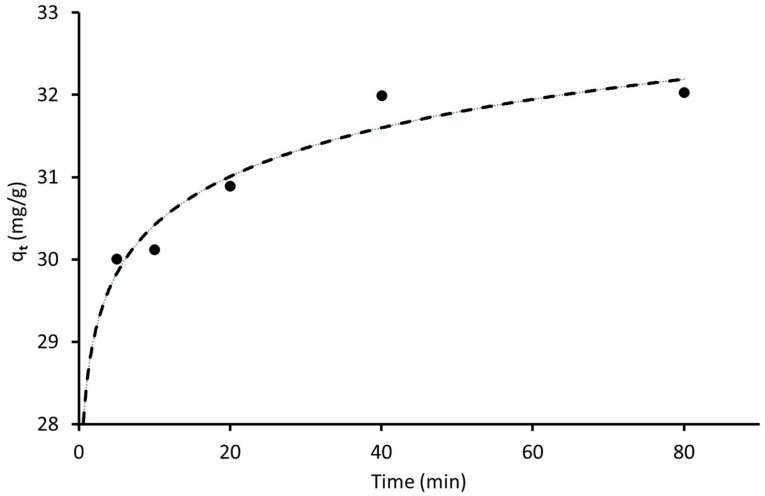
Adsorption capacity of MB against time for SG-EG.

Since the SG-EG sample has shown good adsorption properties towards MB removal and could be considered as a cheaper adsorbent compared with silica gel for the treatment of dye-contaminated wastewater, kinetic studies were performed for the data collected using this sample. Figure 10 shows the change in the MB amount adsorbed by the sample as a function of time and indicates that the adsorption of adsorbate was faster in the first 20 min. A further adsorbed quantity increases more slowly as the surface becomes saturated with the adsorbed molecules. It can be seen from Figure 10 that the time required to reach the adsorption equilibrium between the SG-EG and pollutant was around 40 min.

**Figure 11:**
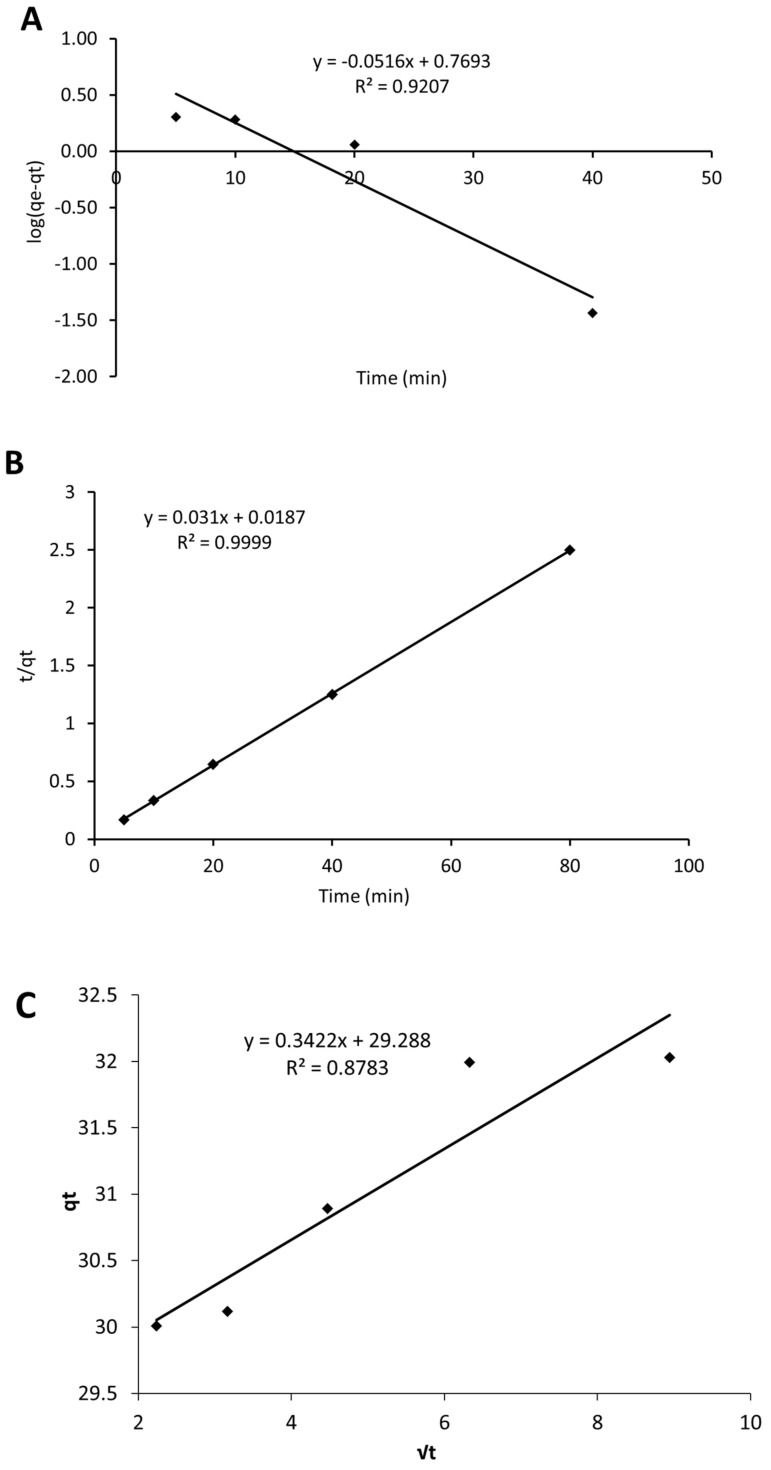
Pseudo-first-order plot (**A**), pseudo-second-order plot (**B**), intra-particle diffusion plot (**C**) for MB adsorption onto SG-ES mixture.

Different kinetic models can be used to analyse the experimental adsorption data from the solutions [80]. In this study, pseudo-first-order, pseudo-second-order and Weber–Morris intra-particle diffusion methods were applied [13,29,81]. The experimental data were fitted to Equations (3)–(5). The corresponding graphical representations are displayed in Figure 11A–C. The parameter values obtained from the applied kinetic models are summarized in Table 4. 

The superiority of the pseudo-second-order model over the pseudo-first-order version results from its excellent correlation coefficient (R^2^ = 0.999 for second order, R^2^ = 0.843 for first order). The better fit of the pseudo-second-order model in a similar adsorbate–adsorbent system had also been reported earlier [29,75]. Moreover, the value of q_e cal_ for SG-EG was slightly higher compared with the experimental value obtained. The q_t_ against t^1/2^ plot corresponding to the Weber–Morris model (Equation (5)) reveals a poor fit; moreover, the curve does not pass through the origin. Therefore, intra-particle diffusion is not the rate-controlling step. The I value is an indication of the occurrence of the boundary layer effect during sorption. The larger the intercept, the greater the contribution of the surface sorption in the rate-controlling step [81]. Thus, the obvious rate-limiting step is the surface sorption of MB onto the SG-ES (Figure 11C). A similar behaviour has been reported for MB removal onto bone char [61].

**Figure 12:**
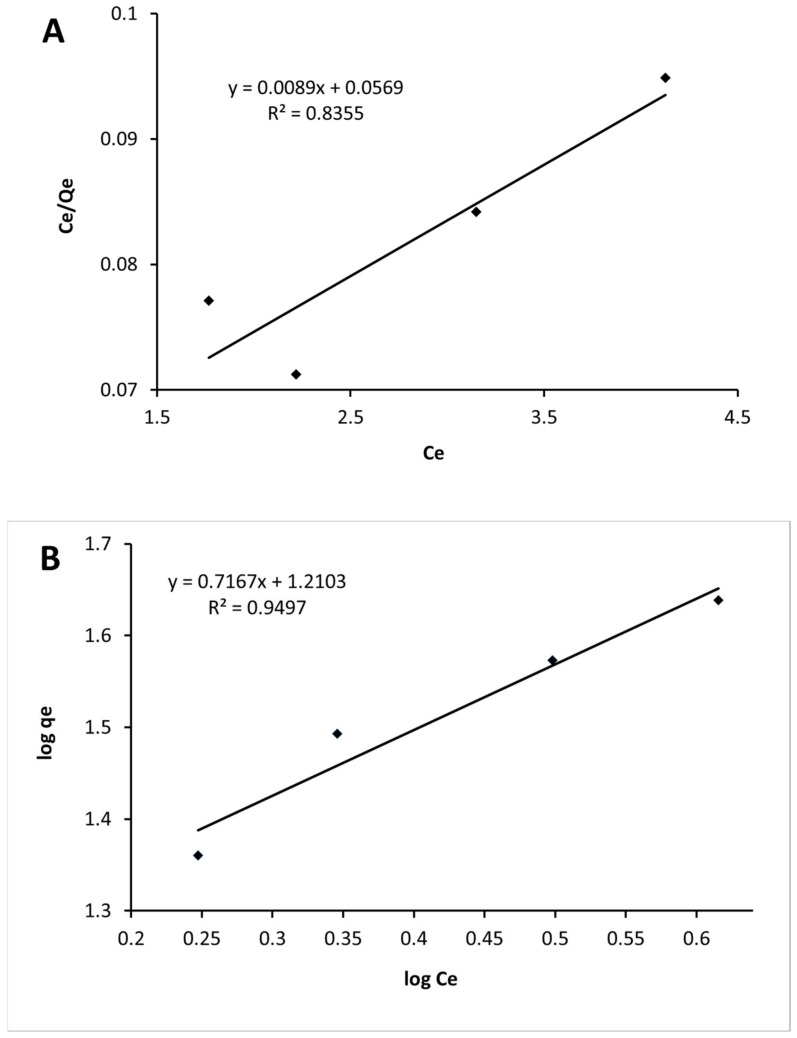
Langmuir isotherm models for MB adsorption on SG-ES mixture (**A**); Freundlich isotherm models for MB adsorption on SG-ES mixture (**B**).

The validity of the Langmuir and Freundlich isotherms for MB was checked by plotting C_e_/q_e_ versus C_e_ (Equation (6)) and log q_e_ versus log C_e_ (Equation (7)), respectively, displayed in Figure 12A,B. The isotherm data were fitted better by the Freundlich equation, with a correlation coefficient (R^2^) of 0.9949 (Figure 12B, Table 5), indicating that the adsorption of MB was a heterogeneous process, and that the monolayer adsorption predicted by Langmuir model was not a reliable option.

Freundlich n indicates the intensity of adsorption and has a value of 1.39. If the value for n > 1, the adsorption is a favourable physical process [82]. Abdel-Khalek et al. [38] studied the adsorption of methylene blue on the surface of eggshell and confirmed that the adsorption isotherm follows the Freundlich model and exhibits multilayer adsorption.

Different possible adsorption mechanisms have been suggested to help gain an understanding of how dye molecules and solid adsorbents interact in an aqueous solution. These include the electrostatic interaction, hydrogen bonding, ion coordination, acid–base interaction, etc. [29,75,83]. Taking into consideration that MB is a cationic organic molecule, it is mostly electrostatically attached near to the anionic hydroxyl groups from the silica surface. According to the literature, above the pH value of 2 in aqueous solutions, the silica surface develops a negative charge due to deprotonation of the silanol Si–O–H groups [84]. In our study, the pH value of the dye solution was set to 7; therefore, the presence of Si–O^−^ groups on SG and SG-containing adsorbents was assumed. The eggshell surface also participates in the adsorption process of the dye. The Ca^2+^, H^+^, HCO_3_^−^, CO_3_^2−^ and OH^−^ ions are reported to be formed at the calcite–water interface [38,74]. The population of those groups are affected by the pH of the solution. The negative charge ions on the eggshell’s solid surface attract the adsorption of MB cations. The excess of negative species occurs at the solution’s low pH values [74]. As reported in the literature, the eggshell and eggshell membrane system showed the maximum adsorption capacity for MB at a pH range from 6 to 8 [38]. The presence of hydroxyl groups, confirmed by FTIR analysis (Figure 7), on the adsorbent mixture surface might also participate in MB molecule bonding. In addition, the weak electrostatic attraction between MB molecules and SG-EG is supported by the Freundlich isotherm model, which is discussed above.

The regeneration process of used samples has been not studied. However, silica-based adsorbent materials can be effectively regenerated due to their mechanical stability. The adsorbed methylene blue can be removed from a silica–sand porous structure by using an alkaline solution (0.1 M NaOH) followed by drying at 60 °C. In the literature, six adsorption–regeneration processes were studied, showing an 18% reduction in sample capacity towards MB adsorption [37].

The regeneration of silica gel is usually performed at relatively at low temperatures between 90 and 150 °C [85]. At this temperature, the active silanol group (−Si-OH) structure can be preserved to a high extent [48]. Those groups have an impact on the thermal degradation process of silica gel [86].

The thermo-analytical investigation result for SG-ES (Figure 8E) supports the above information. No thermal structural decomposition of the SG-ES sample occurred up to 200 °C. During thermal treatment between 20 and 200 °C, the dehydration of the hygroscopic silica gel, resulting in a loss of surface adsorbed water, took place. 

## 4. Conclusions

Within this work, the adsorption of MB from water using prepared different low-cost and available materials, such as silica gel (SG) from desiccant packets, sand from Iraq (SI), eggshell (ES) and their mixtures, was studied. The prepared sorbents were successfully prepared and characterized. The EDX analysis showed that SG and SI have high SiO_2_ contents: 99.2% and 97.5%, respectively. Furthermore, the ES sample’s most abundant component was calcium carbonate (converted as 94.3% CaCO_3_ by TG analysis). 

The low-temperature nitrogen adsorption BET analysis revealed that the porosity and specific surface areas of the mixtures SG-SI and SG-EG increased by a significant extent compared with the sand and eggshell single-component measurements (330 and 360 m^2^/g, respectively). SI and ES alone were not suitable adsorbent materials for waste treatment due to the low values of their surface area (1 and 6 m^2^/g, respectively). The values of the specific surface area and free-pore volume had an outmost effect on the adsorbent material effectiveness in the adsorption efficiency. It was noted that with a decrease in surface area and pore volume values, the adsorption efficiency of silica based adsorbents diminished: 86% SG (S_BET_ = 633 m^2^/g) < 80% SG-ES (S_BET_ = 360 m^2^/g) < 57 %SG-SI (S_BET_ = 330 m^2^/g)

The best behaviour among the mixed samples was noticed for SG-EG. In this system, the MB 10 mg/L removal from aqueous solutions reached the equilibrium within 40 min. The SG-ES mixture produced the highest dye removal yields per mass unit (RE = 80%; q_t_ = 32 mg/g; m = 10 mg). The RE value of 86% for pure silica gel does not bring a significant economic benefit, since at around 50% weight, the eggshell allows the use of an easily available and cheaper adsorbent price. Therefore, the silica gel–eggshell mixture is recommended as a possible adsorbent for the removal of cationic dye from wastewater. 

According to our kinetic studies, we concluded that the MB adsorption results for water on SG-ES excellently fitted the pseudo-second-order adsorption model. The data fitting to the Langmuir and Freundlich isotherms indicated that the Freundlich model fitted the data well, allowing us to conclude that the adsorption does not occur in the monolayer and is not a homogenous process.

It is proposed that the hydrogen bonding and electrostatic interaction occurred between the SG-EG adsorbent surface and cationic MB molecules during the treatment process. Based on TG analysis, the newly prepared SG-EG adsorbents are stable up to 200 °C; therefore, a regeneration temperature under 200 °C is recommended. 

## Figures and Tables

**Table 1:** Composition of studied samples by EDX.

	Sample	SiO_2_, wt%	Al_2_O_3_, wt%	Na_2_O, wt%	CaCO_3_, wt%	SO_3_, wt%	MgO, wt%
1	SI	97.42	2.50	0.08	-	-	-
		97.56	2.4	0.04	-	-	-
	average	97.49	2.45	0.06	-	-	-
2	SG	99.14	0.74	0.12	-	-	-
		99.25	0.75	-	-	-	-
	average	99.20	0.75	0.12	-	-	-
3	EG	0.22	-	-	99.11	0.30	0.36
		0.19	-	-	98.53	0.82	0.46
	average	0.21			98.82	0.56	0.41
4	SG-SI	98.04	1.93	0.03	-	-	-
		98.15	1.81	0.03	-	-	-
	average	98.10	1.87	0.03			
5	SG-EG	30.93	0.13	0.10	68.54	0.17	0.14
		44.82	0.22	0.04	54.63	0.11	0.17
	average	37.88	0.17	0.07	61.58	0.14	0.16

**Table 2:** The surface area, pore volume and pore size of samples studied.

Sample	S_BET,_ m^2^/g	S_micro_, m^2^/g	V, cm^3^/g	V_micro_, cm^3^/g	D_av_, nm
SI	6	0.4	0.0390	0.0002	28.1
ES	1	0	0.0194	0	35.3
SG	633	91	0.2262	0.0485	2.3
SG-ES	360	68	0.1415	0.0279	3.1
SG-SI	330	59	0.1165	0.0246	2.8

**Table 4:** Comparison of the kinetic model equations on the sorption of MB from solution on to SG-EG.

	Pseudo-First Order			Pseudo-Second Order			Intra-Particle Diffusion		
q_e exp_ (mg/g)	k_1_ (min^−1^)	q_e cal_ (mg/g)	R^2^	k_2_ (g/mg min)	q_e cal_ (mg/g)	R^2^	K_d_ (mg/g min^1/2^)	I	R^2^
32.03	0.1188	5.88	0.9207	0.0514	32.26	0.9999	0.3422	29.988	0.8783

**Table 5:** Correlation coefficients and constant parameters calculated for various adsorption models.

Langmuir			Freundlich			
q_max_ (mg/g)	b (L/mg)	R^2^	K_f_ (mg/g) (mg/L)	1/n	n	R^2^
112.36	0.1564	0.8355	16.23	0.72	1.39	0.9497

## Data Availability

Not applicable.

## Supplementary Materials

The following supporting information can be downloaded at: https://www.mdpi.com/article/10.3390/ma16041618/s1, Figure S1: EDX records of adsorbent material studied; Figure S2: FTIR-ATR spectra of the SG-SI (A) and SG-ES (B) adsorbent mixtures and their constituents in the 1300/1900–400 cm^−1^ spectral region; Table S1: Data from thermo-analytical measurements.
