# Supplementary material for: Removal of Methylene Blue from Aqueous Solution by Mixture of Reused Silica Gel Desiccant and Natural Sand or Eggshell Waste
Source: Materials (Basel). 2023 Feb 15;16(4):1618. doi: 10.3390/ma16041618 (PMC9965102; doi:10.3390/ma16041618)
Supplement: Supplementary file 1 [file materials-16-01618-s001.zip › materials-2168563-supplementary.pdf]

Supplementary Materials

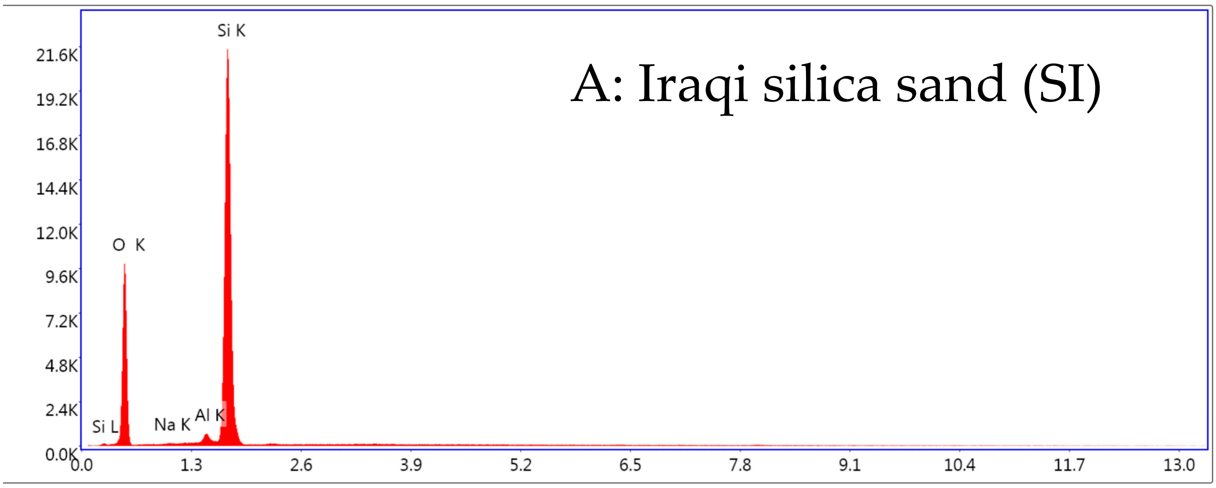

Lsec: 8.5 0 Cnts 0.040 keV Det: Octane Elect Plus

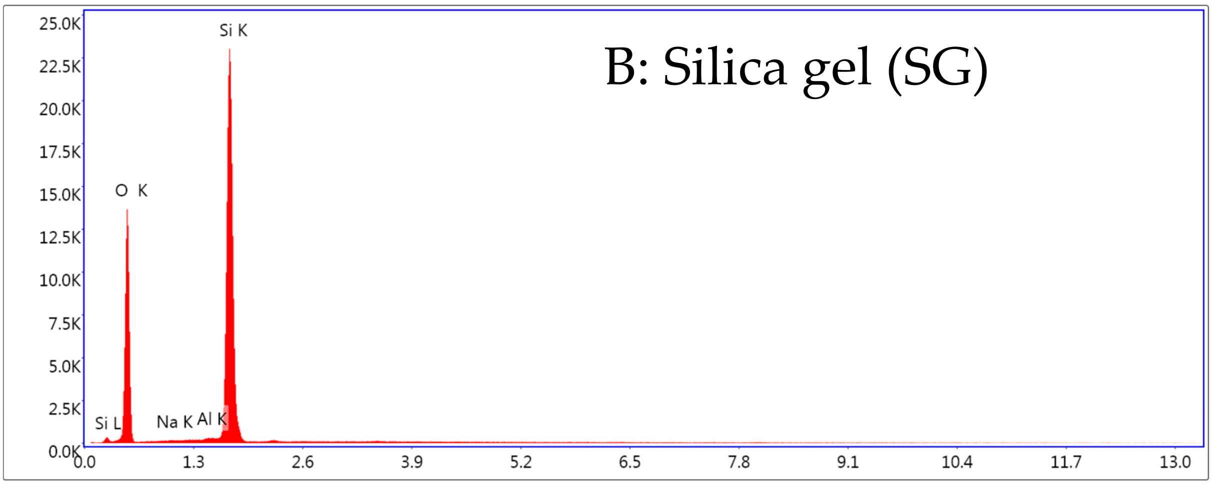

Lsec: 9.3 145 Cnts 2.310 keV Det: Octane Elect Plus

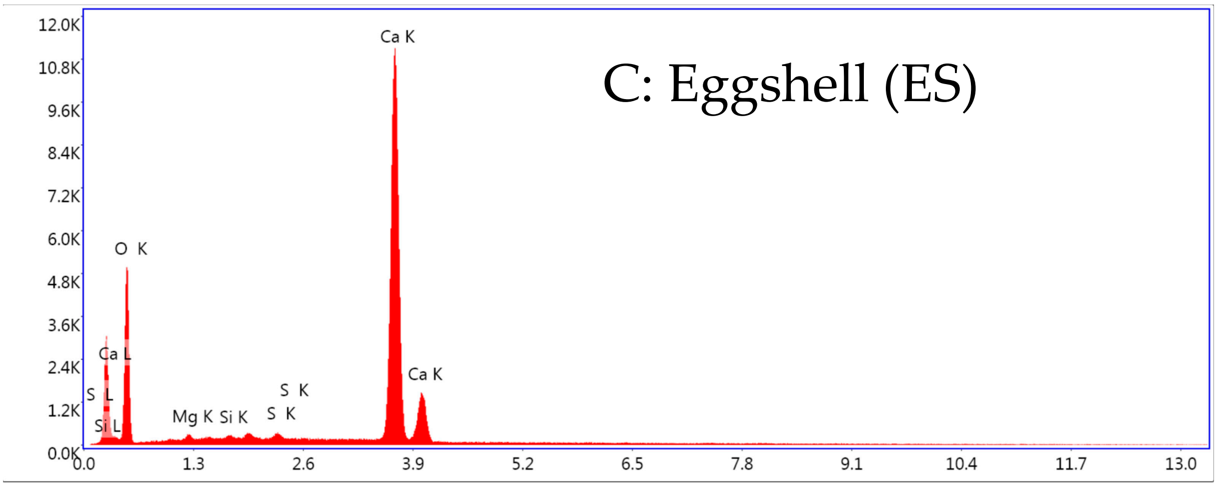

Lsec: 9.9 0 Cnts 0.010 keV Det: Octane Elect Plus

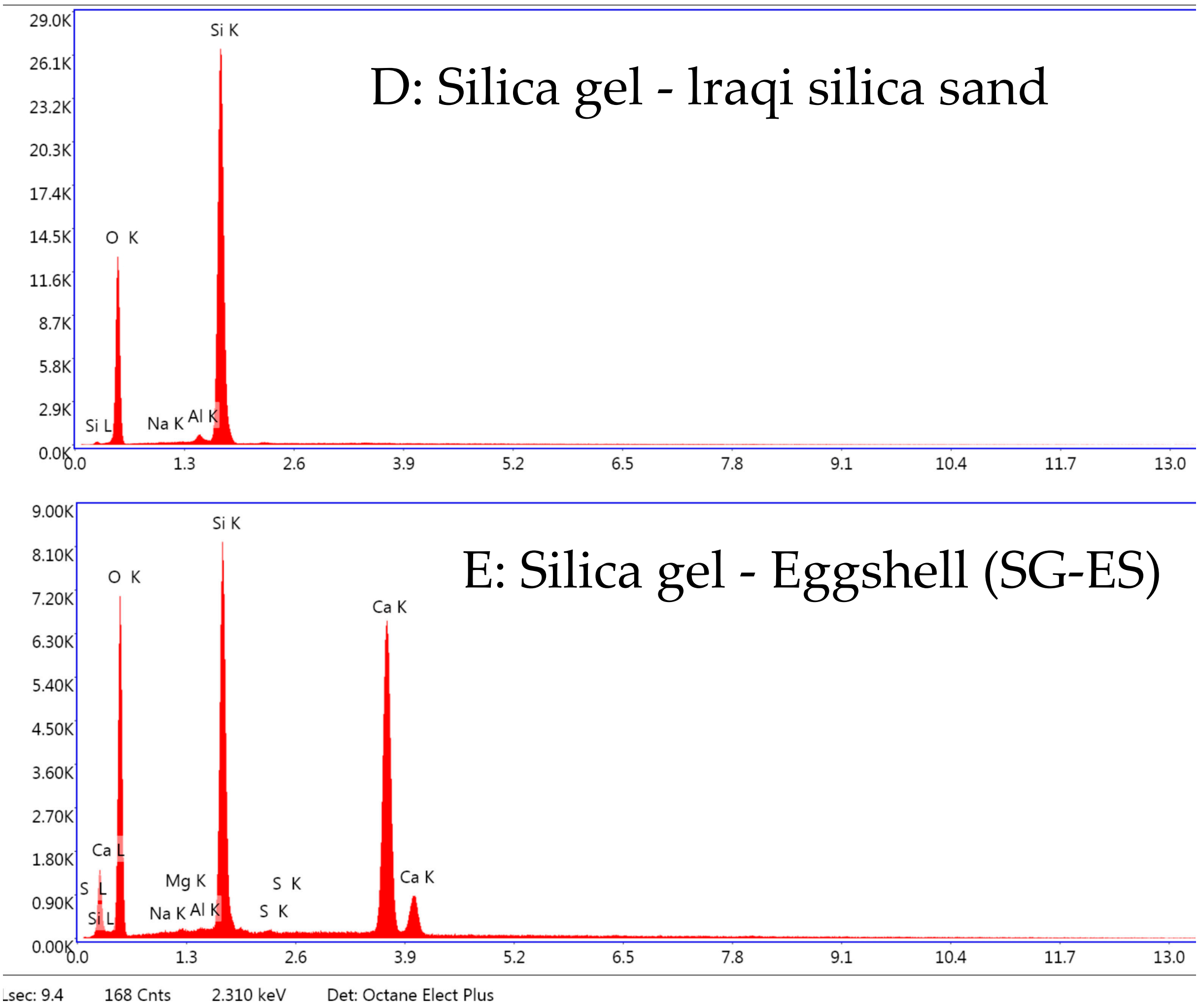

Figure S1. EDX records of adsorbent material studied.

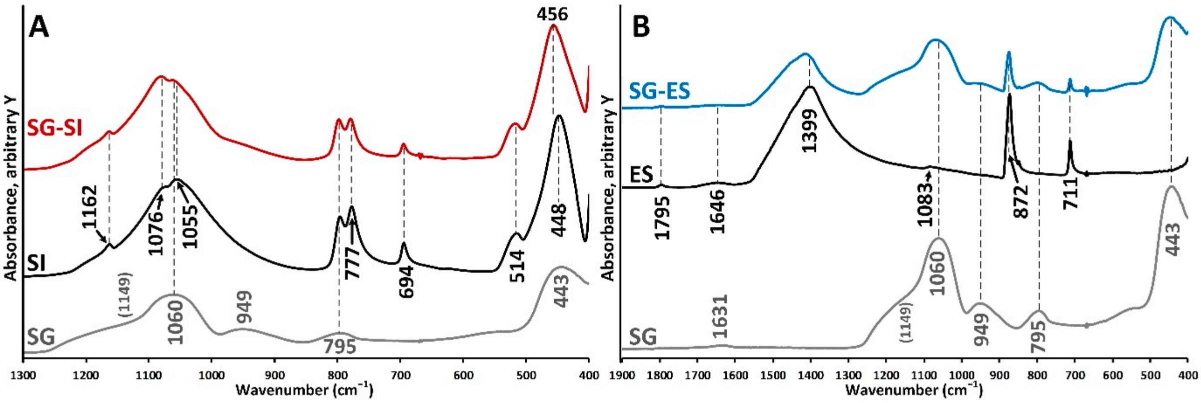

Figure S2. FTIR-ATR spectra of the SG-SI (**A**) and SG-ES (**B**) adsorbent mixtures and their constituents in the 1300/1900–400 cm^{-1} spectral region.

Table S1. Data from thermo-analytical measurements.

Sample	Stage	$T_{initial}$ (°C)	T_{end} (°C)	Mass Loss Step	DTA Signal
**Silica sand** Figure 8A.	I.	20	200	0.2 mg (0.2%)	-
	II.	200	500	0.3 mg (0.3%)	-
	III.	500	720	0.1 mg (0.1%)	-
	IV.	720	1000	0.2 mg (0.2%)	-
	**Initial mass:** 100.9 mg **Total mass loss:** 0.81 mg (0.8%)				
**Silica gel** Figure 8B.	I.	22	265	8.1 mg (8%)	endothermic
	II.	265	800	5 mg (5%)	endothermic
	III.	800	1000	0.8 mg (0.8%)	-
	**Initial mass:** 100.7 mg **Total mass loss:** 13.94 mg (13.8%)				
**Eggshell** Figure 8C.	I.	21	200	0.4 mg (0.4%)	endothermic
	II.	200	480	3.2 mg (3.2%)	exothermic
	III.	480	670	1.0 mg (1.0%)	(endothermic)
	IV.	670	1000	41.7 mg (41.3%)	endothermic
	**Initial mass:** 100.9 mg **Total mass loss**: 46.31 mg (45.9%)				
**Silica sand/Silica gel** Figure 8D.	I.	20	240	2.7 mg (2.7%)	endothermic
	II.	240	790	2.9 mg (2.9%)	(endothermic)
	III.	790	1000	0.5 mg (0.5%)	-
	**Initial mass:** 101 mg **Total mass loss:** 6.16 mg (6.1%)				
**Eggshell/Silica gel** Figure 8E.	I.	20	200	1.4 mg (1.4%)	endothermic
	II.	200	480	2.8 mg (2.8%)	exothermic
	III.	480	670	1.9 mg (1.9%)	(endothermic)
	IV.	670	1000	21.3 mg (21.2%)	endothermic
	**Initial mass:** 100.6 mg **Total mass loss:** 27.46 mg (27.3%)				

SI-Y1 - Calculation of $CaCO_3$ content of the Eggshell sample based on results of thermal analysis

Initial sample mass: 100.9 mg

Adsorbed water content: 0.4 mg (21–200°C)

Dry sample: 100.5 mg

Mass loss observed for the thermal decomposition of calcite: 41.67 mg (670–1000°C)

$$CaCO_3 \rightarrow CO_2 + CaO$$

According to stoichiometry 94.78 mg $CaCO_3$ is required for the liberation of 41.67 mg CO_2

Calculated $CaCO_3$ content can be given relative to the dry sample mass: 94.32%

SI-Y3 - Calculation of $CaCO_3$ content of the Eggshell/Silica gel sample based on results of thermal analysis

Initial sample mass: 100.6 mg

Adsorbed water content: 1.4 mg (21-200°C)

Dry sample: 99.2 mg

Mass loss observed for the thermal decomposition of calcite: 21.33 mg (670-1000°C)

$$CaCO_3 \rightarrow CO_2 + CaO$$

According to stoichiometry 48.51 mg $CaCO_3$ is required for the liberation of 21.33 mg CO_2

Calculated $CaCO_3$ content can be given relative to the dry sample mass: 48.90%

Since the $CaCO_3$ content of the dry Eggshell sample was found to be 94.32% (**SI-Y1**), therefore 48.90% of calcite is expected to be found if 51.85% of the dry sample is constituted of eggshell.
